# Prevalence and associated factors of pre‐hypertension and hypertension in Nepal: Analysis of the Nepal Demographic and Health Survey 2016

**DOI:** 10.1002/hsr2.83

**Published:** 2018-08-10

**Authors:** Gulam Muhammed Al Kibria, Krystal Swasey, Atia Sharmeen, Muhammad Nazmus Sakib, Vanessa Burrowes

**Affiliations:** ^1^ Department of Epidemiology and Public Health, School of Medicine University of Maryland Baltimore Baltimore MD USA; ^2^ School of Community Health and Policy Morgan State University Baltimore MD USA; ^3^ Impulse Hospital Dhaka Bangladesh; ^4^ Bloomberg School of Public Health Johns Hopkins University Baltimore MD USA

**Keywords:** determinants, hypertension, NDHS 2016, Nepal, pre‐hypertension, prevalence

## Abstract

**Objectives:**

Hypertension is the leading risk factor for cardiovascular diseases and develops faster among pre‐hypertensive individuals. However, there is a lack of nationally representative studies that investigate the prevalence and determinants of these two conditions in many developing countries, including Nepal. This study investigates the prevalence and determinants of pre‐hypertension and hypertension in Nepal.

**Methods:**

The present cross‐sectional analysis used data from the 2016 Nepal Demographic and Health Survey, collected from June 2016 to January 2017. After calculating the weighted prevalence (with 95% confidence interval [CI]), simple and multivariable analyses were performed to estimate odds ratios.

**Results:**

A total of 14 857 individuals (6247 males and 8610 females) aged ≥15 years who had their blood pressure measured during the survey were included in this study. The prevalence for pre‐hypertension and hypertension were 26.0% (95% CI: 25.3‐26.3, *n* = 3856) and 19.5% (95% CI: 18.8‐20.2, *n* = 2899), respectively. The prevalence of both conditions was greater among males. In multivariable analyses, older age, male sex, higher body mass index, and residents of Provinces 4 and 5 had significantly increased odds of pre‐hypertension and hypertension (*P* < .05). Additionally, higher education level was found to be positively associated with hypertension.

**Conclusions:**

The combined higher prevalence of pre‐hypertension and hypertension indicates that nearly half (45.5%) of the respondents are at a greater risk of cardiovascular and other non‐communicable diseases due to these two conditions. Older people, males, obese people, and individuals living in Provinces 4 and 5 require more awareness to control blood pressure levels.

**Abbreviations**
AOR: Adjusted odds ratio
BMI: Body mass index
CI: Confidence interval
DBP: Diastolic blood pressure
EA: Enumeration area
NDHS: Nepal Demographic and Health Survey
OR: Odds ratio
SBP: Systolic blood pressure
UOR: Unadjusted odds ratio

## 1 INTRODUCTION

Cardiovascular diseases are currently the leading causes of global deaths or disability‐adjusted life years.^1^, ^2^, ^3^ Hypertension is the principal risk factor for these diseases.^3^, ^4^, ^5^ In addition, hypertension develops faster among pre‐hypertensive persons or people with “high normal” blood pressure.^6^ Over the last few decades, the prevalence of hypertension and other non‐communicable diseases has increased at an alarming rate in developing countries due to the epidemiologic and demographic transitions in these countries.^2^, ^3^ Moreover, many developing countries are currently dealing with a problem that is also known as “twin” or “double” disease burden, where there is a concomitant higher incidence and prevalence of communicable and non‐communicable diseases.^7^, ^8^, ^9^ Although there have been continuous surveillance efforts to monitor incidence, trends, and prevalence of both communicable and non‐communicable diseases in developed countries, limited information is available from developing countries. Most of the estimates in developing countries come from population‐based or small‐scale cross‐sectional studies that are designed to estimate the overall burden and determinants of diseases.^1^, ^2^, ^3^ Among the regions of the World Health Organization (WHO), hypertension and other cardiovascular disorders are increasing at a faster rate in South Asian countries compared with other regions.^7^, ^8^, ^9^ Previous studies reported that prevalence and likelihood of pre‐hypertension and hypertension vary according to several characteristics, including age, sex, body mass index (BMI), socioeconomic status, and place of residence.^10^, ^11^, ^12^ These characteristics also affect awareness, treatment, and control of hypertension.^11^, ^12^

Nepal is a South Asian developing country with an estimated population of 29 million people, residing in a land mass of 147 181 square kilometers. This country is divided into seven provinces (Provinces 1‐7), in three ecological zones: Mountain, Hill, and Terai.^13^ Similar to many other countries, Nepal is facing the twin burden of diseases, and prevalence of hypertension is increasing at an alarming rate.^8^, ^9^ This country also lacks nationally representative data on the prevalence of hypertension. The Nepal Demographic and Health Survey 2016 (2016 NDHS) was one of the few surveys conducted in this country to estimate the overall prevalence of hypertension in Nepal. The prevalence of hypertension among males and females in that survey was 23% and 17%, respectively. In addition, the prevalence of pre‐hypertension was 31% among males and 24% among females.^14^ As the 2016 NDHS reported the prevalence after stratifying according to sex instead of demonstrating the overall prevalence of blood pressure levels according to background characteristics, this limits the understanding of the overall prevalence or burden of hypertension in this country. Additionally, this survey also reported the point estimate of the prevalence of pre‐hypertension or hypertension instead of the 95% confidence interval (CI); it is more important to report the CI to have a more precise estimate.^15^

Studies that previously investigated prevalence and risk factors for hypertension in Nepal and other countries have found that these vary according to age, sex, body weight, race/ethnicity, place of residence, marital status, education level, socio‐economic status, and concomitant diseases such as diabetes, dyslipidemia, or stress.^12^, ^16^, ^17^, ^18^, ^19^, ^20^ Furthermore, due to similar restrictions for assessing prevalence from a nationally representative dataset, studies investigating the determinants of pre‐hypertension or hypertension in Nepal are also limited by a shortage in recently collected data and the availability of only small‐scale studies mainly conducted in a community or a particular region.^7^, ^10^, ^11^, ^16^, ^21^, ^22^, ^23^ Investigating prevalence and determinants from a nationally representative dataset is essential for designing and implementing a national evidence‐based strategy to prevent and control pre‐hypertension and hypertension, as well as minimizing the complications associated with these two conditions in this country.

In this study, with the aim of identifying and filling these existing knowledge gaps, we analyzed the 2016 NDHS data to investigate the “overall” prevalence and determinants of pre‐hypertension and hypertension in Nepal. Our results may also be helpful for estimating this health burden in other South Asian countries with similar socio‐demographic characteristics, double disease burden, and limited availability of recent data.

## 2 METHODS

### 2.1 Data source

New Era, a private research organization in Nepal, conducted the 2016 NDHS from June 2016 to January 2017. This survey had the specific objective of estimating the prevalence of hypertension in this country. This de‐identified dataset was available for academic and scientific use upon approval from the ICF International, Maryland, United States.^14^ We obtained approval to use the data in January 2018. The Nepal Health Research Council and the ICF Institutional Review Board approved the survey protocol. In each surveyed home, the head of household provided written informed consent on behalf of all members.^14^

### 2.2 Survey procedure

The 2016 NDHS was designed to make the survey nationally representative. This was a multistage survey, conducted in two and three stages in rural and urban areas, respectively. The ward was considered as the primary sampling unit in both areas. Then, in rural areas, households were selected from wards. As the wards were larger in urban areas, each ward, according to the older ward classification from the 2011 Nepal Population and Housing Census, was considered as the enumeration area (EA) in the second stage. Again, the households were selected from the EA. The survey aimed to have a total of 11 490 households. From these households, all residents aged ≥15 years were eligible for blood pressure measurements. On behalf of the household members, the head of each household provided written informed consent. With an overall 95% response rate, a total of 14 823 “un‐weighted” individuals participated in the survey. The survey design, methodologies, sample size calculation, findings, and questionnaires are available elsewhere.^14^

### 2.3 Measurements

The survey used the UA‐767F/FAC (A&D Medical) automated device to record the blood pressure of the participants. Blood pressure was measured three times in a sitting position, with a gap of 5 minutes between each measurement, and the mean of the last two measures was used to report the pressure levels. The survey used the World Health Organization 1999 guidelines (1999 WHO) to report a participant as hypertensive.^14^ This guideline is currently recommended by WHO among the existing guidelines and reports, including the 2017 American College of Cardiology/ American Society of Hypertension guidelines.^24^, ^25^

### 2.4 Study variables

The dependent variable for this study was hypertension. A person with a systolic blood pressure (SBP) ≥140 mmHg or a diastolic blood pressure (DBP) ≥90 mmHg was considered hypertensive. Additionally, participants exhibiting values below that pressure range for SBP and DBP but taking blood pressure lowering drugs, were considered hypertensive. An individual with either SBP or DBP between 120 and 139 or 80 and 89 mmHg, respectively, without taking blood pressure lowering drugs, was labeled as prehypertensive. The explanatory variables (ie, factors) were selected a priori based on available reports and structure of the 2016 NDHS. The independent variables were age (in years), sex, BMI (kg/m^2^), education level, household wealth status, place (rural or urban), ecological zone (Mountain, Hill or Terai), and provinces (Provinces 1‐7) of residence. Table 1 describes all study variables and their categories.

**Table 1:** Study variables

Study Variables	Definition	Category
Pre‐hypertension	A person with SBP 120‐139 mm Hg or DBP 80‐89 mm Hg and the person was not taking any blood pressure lowering drugs.	Binary 0 = No; 1 = Yes
Hypertension	An SBP ≥140 mm Hg or DBP ≥90 mm Hg or the person was taking prescribed anti‐hypertensive drugs.	Binary 0 = No; 1 = Yes
Controlled on medication	An SBP ≤140 mm Hg or DBP ≤90 mm Hg and the person was taking any prescribed anti‐hypertensive drugs.	Binary 0 = No; 1 = Yes
Stage 1 hypertension	An SBP 140‐159 mm Hg or DBP 90‐99 mm Hg or the person was taking prescribed anti‐hypertensive drugs with an SBP ≤140 mm Hg and DBP ≤90.	Binary 0 = No; 1 = Yes
Stage 2 hypertension	An SBP ≥160 mm Hg or DBP ≥100 mm Hg.	Binary 0 = No; 1 = Yes
Age	Age of the respondents in years.	Ordinal 1 = 20‐34; 2 = 35‐44; 3 = 45‐54; 3 = 55‐64; 4 = ≥65
Sex	Sex of the respondents.	Binary 1 = Male; 2 = Female
Body mass index (BMI)	BMI of the respondents (kg/m^2^). Obtained by dividing weight (in kilograms) with square of the height (in meters).	Ordinal 0 = <18.5; 1 = 18.5‐24.9 2 = 25‐29.9; 3 = ≥30.
Education	Education level of the respondents.	Ordinal 0 = No formal education; 1 = Primary (1‐5 years); 2 = Secondary (6‐10 years) or higher
Household wealth status	Composite index of household materials; obtained by principal component analysis.	Ordinal 1 = Poorest; 2 = Poorer; 3 = Middle; 4 = Richer; 5 = Richest.
Place of residence	Whether the person is living in a rural or an urban area at the time of interview.	Binary 1 = urban; 2 = Rural
Ecological zone	Ecological zone where the person was living.	Nominal 1 = Mountain; 2 = Hill; 3 = Terai
Province	Province of residence. Province is the largest administrative unit in this country.	Nominal 1 = Province 1; 2 = Province 2; 3 = Province 3; 4 = Province 4; 5 = Province 5; 6 = Province 6; 7 = Province 7

### 2.5 Statistical analyses

First, the background characteristics of the study participants were described. Continuous variables with skewed distributions were reported with median and inter‐quartile ranges (IQR). Categorical variables were reported with numbers and percentages. Prevalence of hypertension and pre‐hypertension was determined according to the background characteristics of the study participants. Then, we conducted simple logistic regression analyses to estimate the unadjusted odds ratios (UOR) with 95% CI. Next, a multivariable analysis was conducted to estimate the adjusted odds ratio (AOR). Variance inflation factors were assessed before incorporating them into the multivariable models. Variables with a *P* value <0.2 in simple logistic regression were considered in the multivariable model to estimate the AOR. Stata 14.0 (Stata Corporation, TX, USA) was used to analyze data in this study. The overall proportion of missing data was less than 2%; we, therefore, did not impute for any missing data. We considered the hierarchical structure of the 2016 NDHS dataset after accounting for cluster sampling design of the 2016 NDHS to estimate the prevalence and determinants in this study.^14^

## 3 RESULTS

Table 2 shows the background characteristics of the study participants. Of the total 14 857 participants included in this study, 8102, 3856, and 2899 had normal blood pressure, pre‐hypertension, and hypertension, respectively. The overall median SBP and DBP were 112 (IQR: 103‐124) mm Hg and 77 (IQR: 70‐84) mm Hg, respectively. For hypertensive people, the median SBP was 141 (IQR: 129‐154) mm Hg, and the median DBP was 93 (IQR: 89‐99) mm Hg. Hypertensive participants were older (median: 51, IQR: 38‐62 years) than their pre‐hypertensive (median: 38, IQR: 26‐52 years) or normotensive (median: 28, IQR: 20‐43 years) counterparts. The percentage of the overall population and hypertensive people who were taking blood pressure lowering medication were 3.9% and 20.5%, respectively (*n* = 585 for both). About half of the participants (48.6%) were below 35 years of age. Although the proportion of people ≥65 years of age was 10.0% (1483/14 857), this proportion was 22.0% (*n* = 638) among hypertensive persons (*n* = 2889).

**Table 2:** Background characteristics of the survey participants

Characteristics^a^	Normal Pressure, (*n* = 8102)	Pre‐Hypertension, (*n* = 3856)	Hypertension, (*n* = 2899)	Overall, (*N* = 14 857)^b^
SBP, median (IQR), mm Hg	104 (98‐110)	120 (114‐126)	141 (129‐154)	112 (103‐124)
DBP, median (IQR), mm Hg	71 (66‐75)	82 (80‐85)	93 (89‐99)	77 (70‐84)
Take anti‐hypertensive	‐‐	‐‐	585 (20.2)	585 (3.9)
Age, years				
Median (IQR)	28 (20‐43)	38 (26‐52)	51 (38‐62)	35 (23‐31)
15‐24	3187 (39.3)	762 (19.8)	154 (5.3)	4103 (27.6)
25‐34	1928 (23.8)	845 (21.9)	351 (12.1)	3124 (21.0)
35‐44	1178 (14.5)	792 (20.5)	569 (19.6)	2505 (17.1)
45‐54	786 (9.7)	623 (16.2)	607 (20.9)	2016 (13.6)
55‐64	565 (7.0)	447 (11.6)	579 (20.0.)	1591 (10.7)
≥65	458 (5.6)	387 (10.1)	638 (22.0)	1483 (10.0)
Sex				
Male	1923 (36.1)	1861 (48.2)	1463 (50.4)	6247 (42.0)
Female	5179 (63.9)	1995 (51.8)	1436 (49.6)	8610 (58.0)
Body mass index, kg/m^2^				
Median (IQR)	20.4 (18.6‐22.6)	21.9 (19.8‐24.6)	23.1 (20.4‐26.3)	21.2 (19.1‐23.9)
<18.5	1877 (23.4)	519 (13.6)	316 (11.1)	2712 (18.5)
18.5‐24.9	5193 (64.6)	2382 (62.4)	1492 (52.6)	9067 (61.7)
25‐29.9	818 (10.2)	746 (19.6)	789 (27.8)	2354 (16.0)
≥30	150 (1.9)	168 (4.4)	237 (8.4)	555 (3.8)
Education				
No formal education	2689 (33.2)	1529 (39.6)	1372 (47.4)	5590 (37.6)
Primary	1278 (15.8)	659 (17.1)	529 (18.3)	2466 (16.6)
Secondary or above	4136 (51.0)	1666 (43.2)	996 (34.4)	6798 (45.8)
Wealth quintile				
Poorest	1426 (17.6)	776 (20.1)	463 (16.0)	2666 (17.9)
Poorer	1552 (19.2)	765 (19.9)	565 (19.5)	2882 (19.4)
Middle	1744 (21.5)	747 (19.4)	486 (16.8)	2978 (20.0)
Richer	1884 (23.2)	811 (21.0)	564 (19.5)	3259 (21.9)
Richest	1495 (23.3)	755 (19.6)	821 (28.3)	3071 (20.7)
Place of residence				
Rural	4915 (60.7)	2326 (60.3)	1871 (64.6)	9112 (61.3)
Urban	3187 (39.3)	1530 (39.7)	1028 (35.4)	5744 (38.7)
Ecological zone				
Mountain	549 (6.8)	240 (6.2)	162 (5.6)	951 (6.4)
Hill	3280 (40.5)	1802 (46.7)	1459 (50.4)	6542 (44.0)
Terai	4274 (52.7)	1813 (47.0)	1277 (44.1)	7365 (49.6)
Division				
Province 1	1471 (18.2)	656 (17.0)	493 (17.0)	2620 (17.6)
Province 2	1918 (23.7)	658 (17.1)	454 (15.7)	3031 (20.4)
Province 3	1644 (20.3)	849 (22.0)	745 (25.7)	3239 (21.8)
Province 4	659 (8.1)	466 (12.1)	408 (14.1)	1533 (10.3)
Province 5	1189 (14.7)	716 (18.6)	519 (17.9)	2424 (16.3)
Province 6	461 (5.7)	190 (4.9)	111 (3.8)	762 (5.1)
Province 7	758 (9.4)	319 (8.3)	168 (5.8)	1246 (8.4)

a Column percentage unless otherwise specified.

b Numbers may not add up to total because of missing values.

DBP, diastolic blood pressure; IQR, inter‐quartile range; SBP, systolic blood pressure.

The overall percentage of males and females in the study were 42.0% (*n* = 6245) and 58.0% (*n* = 8211), respectively. About 37.4% of the respondents had no formal education (*n* = 5590). Overall, more than 60% of the people were from urban areas. The highest proportion of the respondents was from Province 3 (21.8%), followed by Provinces 2 (20.4%), 1 (17.6%), 5 (16.3%), 4 (10.4%), 7 (8.4%), and 6 (5.1%).

Table 3 shows the stages of hypertension according to antihypertensive medication use. Among the people who were not on antihypertensive medication (*n* = 14 272), the prevalence of pre‐hypertension and hypertension was 27.0% (95% CI: 26.3‐27.7) and 16.2% (95% CI: 15.6‐16.8), respectively. On the other hand, among the respondents who were on anti‐hypertensive medication (*n* = 585), nearly half had uncontrolled hypertension, 47.8% (95% CI: 43.8‐51.8). The overall prevalence of “stage 1” and “stage 2” hypertension was 13.5% (95% CI: 12.9‐14.0) and 6.0% (95% CI: 5.6‐6.4), respectively.

**Table 3:** Prevalence (95% confidence interval) of blood pressure stages according to anti‐hypertensive drug use^a^

Blood Pressure Level	Not on Anti‐Hypertensive Drugs (*n* = 14 272)	On Anti‐Hypertensive Drugs (*n* = 585)	Overall (*n* = 14 857)
Normal/controlled on medication^b^	56.8 (55.9‐57.6)	19.1 (16.1‐22.5)	54.5 (53.7‐55.3)
Pre‐hypertension/controlled on medication^b^	27.0 (26.3‐27.7)	33.1 (29.4‐37.0)	26.0 (25.3‐26.7)
Stage 1 hypertension	11.0 (10.5‐11.5)	22.6 (19.3‐26.1)	13.5 (12.9‐14.0)
Stage 2 hypertension	5.2 (4.9‐5.6)	25.2 (21.9‐28.9)	6.0 (5.6‐6.4)
Crude hypertension	16.2 (15.6‐16.8)	47.8 (43.8‐51.8)	19.5 (18.9‐20.2)

a Column percentage.

b “Controlled on medication” only for the people who were on antihypertensive medication.

Table 4 shows the prevalence (with 95% CI) of pre‐hypertension and hypertension according to the background characteristics of the study participants. The overall prevalence of pre‐hypertension and hypertension was 26.0% (95% CI: 25.3‐26.3) and 19.5% (95% CI: 18.8‐20.2), respectively. The prevalence of pre‐hypertension, across characteristics, ranged from 18.6% to 31.8%. Prevalence of hypertension increased with participant age, with the lowest value among participants 15 to 24 years of age, 3.8% (95% CI: 3.2‐4.4), and the highest among those ≥65 years of age, 43.1% (95% CI: 40.1‐45.6). Prevalence of hypertension according to BMI also showed an ordinal pattern, with the highest, 42.7% (95% CI: 38.6‐46.8), among the people with the highest BMI (≥30 kg/m^2^). Females had a lower prevalence of hypertension than their male counterparts, 16.7% (95% CI: 15.9‐17.5) and 23.4% (95% CI: 22.4‐24.5), respectively. Pre‐hypertension had a similar pattern in prevalence, 23.2% (95% CI: 22.3‐24.1) and 29.8% (95% CI: 28.7‐30.9) among females and males, respectively. People with no formal education had a greater prevalence of pre‐hypertension and hypertension compared with people with any type of formal education. The prevalence of hypertension was 20.5% (95% CI: 19.7‐21.4) in rural areas, slightly higher than that in urban areas, at 17.9% (95% CI: 16.9‐18.9). Province 4 had the highest prevalence of pre‐hypertension and hypertension, 30.4% (95% CI: 28.1‐32.7) and 26.6% (95% CI: 24.4‐28.9), respectively.

**Table 4:** Prevalence (95% confidence interval) of pre‐hypertension and hypertension according to background characteristics^a^

Characteristics	Pre‐Hypertension	Hypertension
Age group, years		
15‐24	18.6 (17.4‐19.8)	3.8 (3.2‐4.4)
25‐34	27.0 (25.5‐28.6)	11.2 (10.2‐12.4)
35‐44	31.2 (29.4‐33.0)	22.4 (20.8‐24.1)
45‐54	30.9 (28.9‐32.0)	30.1 (28.1‐32.2)
55‐64	28.1 (25.9‐30.3)	36.4 (34.1‐38.8)
≥65	26.1 (23.9‐28.4)	43.1 (40.5‐45.6)
Sex		
Male	29.8 (28.7‐30.9)	23.4 (22.4‐24.5)
Female	23.2 (22.3‐24.1)	16.7 (15.9‐17.5)
Body mass index, kg/m^2^		
<18.5	19.1 (17.7‐20.6)	11.7 (10.5‐12.9)
18.5‐24.9	26.3 (25.4‐27.2)	16.5 (15.7‐17.2)
25‐29.9	31.8 (29.9‐33.6)	33.5 (31.6‐35.5)
≥30	30.3 (26.6‐34.3)	42.7 (38.6‐46.8)
Education		
No formal education	27.4 (26.2‐28.5)	24.5 (23.4‐25.7)
Primary	26.7 (25.0‐28.5)	21.5 (19.9‐23.1)
Secondary or above	24.5 (23.5‐25.5)	14.7 (13.8‐15.5)
Wealth quintile		
Poorest	29.1 (27.4‐30.9)	17.4 (16.0‐18.8)
Poorer	26.6 (25.0‐28.2)	19.6 (18.2‐21.1)
Middle	25.1 (23.6‐26.7)	16.3 (15.0‐17.7)
Richer	24.9 (23.4‐26.4)	17.3 (16.0‐18.6)
Richest	24.6 (23.1‐26.1)	26.7 (25.2‐28.3)
Place of residence		
Rural	25.5 (24.6‐26.4)	20.5 (19.7‐21.4)
Urban	26.6 (25.5‐27.8)	17.9 (16.9‐18.9)
Ecological zone		
Mountain	25.3 (22.6‐28.1)	17.0 (14.7‐19.5)
Hill	27.6 (26.5‐28.6)	22.3 (21.3‐23.3)
Terai	24.6 (23.7‐26.2)	17.3 (16.5‐18.2)
Division		
Province 1	25.0 (23.4‐26.7)	18.8 (17.4‐20.4)
Province 2	21.7 (20.3‐23.2)	15.0 (13.8‐16.3)
Province 3	26.2 (24.7‐27.8)	23.0 (21.6‐24.5)
Province 4	30.4 (28.1‐32.7)	26.6 (24.4‐28.9)
Province 5	29.5 (27.8‐31.4)	21.4 (19.8‐23.1)
Province 6	25.0 (22.0‐28.2)	14.5 (12.2‐17.2)
Province 7	25.6 (23.3‐28.1)	13.5 (11.7‐15.5)
Overall	26.0 (25.3‐26.3)	19.5 (18.8‐20.2)

a Row percentage.

The results of logistic regression analyses are summarized in Table 5. Both pre‐hypertension and hypertension had some common associated factors including age, sex, BMI, and province of residence. Age and BMI, two ordinal variables, had “dose‐response” relationships with pre‐hypertension and hypertension. After adjusting for all the variables, people with ≥65 years of age had nearly 4‐fold higher odds of having pre‐hypertension (AOR: 3.7, 95% CI: 3.0‐4.6) and 37‐fold greater odds of having hypertension (AOR: 36.8, 95% CI: 26.7‐50.8). Female sex had an inverse association with hypertension and pre‐hypertension in both bivariate and multivariable analyses (both OR: 0.6). Similarly, living in Provinces 4 or 5 was associated with an increased likelihood of both pre‐hypertension and hypertension in both adjusted and un‐adjusted levels (all OR: 1.6). Primary and secondary education levels were positively associated with hypertension (AOR for both education levels: 1.3, 95% CI: 1.0‐1.6). Wealth status had no significant relationship with hypertension in multivariable analysis, and the place or ecological zone of residence was not significantly associated with either of hypertension or pre‐hypertension.

**Table 5:** Results of logistic regression analyses

Characteristics	Pre‐Hypertension Vs Normal Pressure		Hypertension Vs Normal Pressure	
	UOR (95% CI)	AOR (95% CI)	UOR (95% CI)	AOR (95% CI)
Age group, years				
15‐24	Ref.	Ref.	Ref.	Ref.
25‐34	1.8*** (1.6,2.1)	1.6*** (1.4,1.9)	3.8***(2.9,4.8)	3.0*** (2.3,3.9)
35‐44	2.8*** (2.4,3.3)	2.4*** (2.0,2.9)	10.0***(7.8,12.7)	7.6*** (5.9,10.0)
45‐54	3.3*** (2.7,4.0)	3.0*** (2.4,3.7)	16.0***(12.1,21.1)	14.2*** (10.2,19.8)
55‐64	3.3*** (2.8,3.9)	3.1*** (2.5,3.8)	21.2*** (16.6,27.1)	22.2*** (16.7,29.6)
≥65	3.5***(3.0,4.2)	3.7*** (3.0,4.6)	28.9*** (22.1,37.7)	36.8*** (26.7,50.8)
Sex				
Male	Ref.	Ref.	Ref.	Ref.
Female	0.6*** (0.6,0.7)	0.6*** (0.5,0.6)	0.6*** (0.5,0.6)	0.6*** (0.5,0.7)
Body mass index, kg/m^2^				
<18.5	Ref.	Ref.	Ref.	Ref.
18.5‐24.9	1.7*** (1.5,1.9)	1.7*** (1.5,1.9)	1.7*** (1.5,2.0)	2.0*** (1.6,2.3)
25‐29.9	3.3*** (2.8,3.9)	3.5*** (2.9,4.1)	5.7*** (4.6,7.1)	5.7*** (4.6,7.2)
≥30	4.1*** (2.9,5.7)	4.4*** (3.1,6.5)	9.4*** (6.5,13.5)	9.2*** (6.0,14.0)
Education				
No formal education	Ref.	Ref.	Ref.	Ref.
Primary	0.9 (0.8,1.0)	1.0 (0.8,1.2)	0.8* (0.7,1.0)	1.3* (1.0,1.6)
Secondary or above	0.7*** (0.6,0.8)	1.0 (0.9,1.2)	0.5*** (0.4,0.5)	1.3* (1.0,1.6)
Wealth quintile				
Poorest	Ref.	Ref.	Ref.	Ref.
Poorer	0.9 (0.8,1.0)	0.9 (0.8,1.1)	1.1 (0.9,1.3)	1.1 (0.9,1.4)
Middle	0.8** (0.7,0.9)	0.8* (0.7,1.0)	0.9 (0.7,1.0)	1.0 (0.8,1.3)
Richer	0.8* (0.7,0.9)	0.8* (0.6,1.0)	0.9 (0.7,1.1)	0.9 (0.7,1.2)
Richest	0.9 (0.8,1.1)	0.7** (0.6,0.9)	1.7*** (1.3,2.1)	1.2 (0.9,1.6)
Place of residence				
Rural	Ref.	Ref.	Ref.	Ref.
Urban	1.0 (0.9,1.2)	1.0 (0.8,1.2)	0.8 (0.7,1.0)	0.9 (0.8,1.1)
Ecological zone				
Mountain	Ref.	Ref.	Ref.	Ref.
Hill	1.3 (1.0,1.6)	1.2 (0.9,1.6)	1.5* (1.1,2.1)	1.1 (0.8,1.6)
Terai	1.0 (0.8,1.2)	1.1 (0.8,1.5)	1.0 (0.7,1.4)	1.0 (0.7,1.4)
Province				
Province 1	Ref.	Ref.	Ref.	Ref.
Province 2	0.8* (0.6,1.0)	0.9 (0.7,1.2)	0.7** (0.6,0.9)	0.9 (0.7,1.2)
Province 3	1.2 (0.9,1.5)	1.2 (0.9,1.5)	1.4 (1.0,1.8)	1.2 (0.9,1.7)
Province 4	1.6*** (1.3,2.0)	1.6*** (1.2,2.1)	1.8*** (1.4,2.4)	1.6** (1.2,2.3)
Province 5	1.4** (1.1,1.7)	1.6*** (1.2,2.0)	1.3 (1.0,1.7)	1.6** (1.2,2.2)
Province 6	0.9 (0.7,1.2)	1.0 (0.8,1.3)	0.7* (0.5,1.0)	0.9 (0.6,1.4)
Province 7	0.9 (0.7,1.2)	1.1 (0.8,1.4)	0.7** (0.5,0.9)	0.8 (0.6,1.2)

AOR, adjusted odds ratio; CI, confidence interval; UOR, unadjusted odds ratio.

* *P* value <0.05.

** *P* value <0.01.

*** *P* value <0.001.

## 4 DISCUSSION

In this study, we analyzed a nationally representative survey to estimate the prevalence and associated factors of pre‐hypertension and hypertension in Nepal.^14^ To our knowledge, this is the first nationally representative study from Nepal to present the most recent data (2016 NDHS) on prevalence and risk factors for both pre‐hypertension and hypertension in relation to a range of background characteristics.

The combined prevalence of pre‐hypertension and hypertension in this study was 45.5%—this collective prevalence indicates that nearly half of the people aged ≥15 years in Nepal are above the normal blood pressure level and at a greater risk of cardiovascular diseases due to these conditions.^24^, ^26^ This prevalence is similar to other developing countries as well as developed countries.^4^, ^27^, ^28^, ^29^, ^30^, ^31^ We found an increased prevalence and odds of hypertension and pre‐hypertension among older persons, males, overweight people, and in some specific places of residence. Additionally, people with a higher level of education had a greater likelihood of hypertension.

Of the factors associated with a higher prevalence or likelihood of hypertension in this study, only obesity or overweight (BMI ≥25 kg/m^2^) status was modifiable. This finding is consistent with the results of previous studies conducted in Nepal.^11^, ^16^, ^17^ Obese people have increased risks of hypertension, diabetes, and other non‐communicable diseases.^32^, ^33^, ^34^ This condition could have synergistic effects on negative consequences associated with hypertension.^32^, ^34^ Elevated BMI is an emerging problem not only in developing countries but globally as well.^16^, ^35^, ^36^ Although the overall proportion of obese or overweight respondents was low in this study, modifying dietary habits and lifestyles is essential to reduce the burden of obesity. Such a decrease may help prevent or control hypertension and other diseases that occur as complications of hypertension or obesity.^32^, ^33^, ^34^

Similar to several other countries, our results show that overall prevalence and likelihood of pre‐hypertension or hypertension was higher among males.^37^, ^38^, ^39^ Both biological and behavioral differentials contribute to this major difference.^37^ The 2016 NDHS report stratified prevalence according to sex and found that males had a greater prevalence than their female counterparts until 65 years of age.^14^ These differences suggest that males require more awareness to maintain normal blood pressure levels than females.

In line with some studies conducted in Nepal, the prevalence and risk factors for hypertension or pre‐hypertension did not show any consistent patterns of association with variables of socioeconomic conditions such as education level and wealth status.^10^, ^17^ Although wealth status had no relationship with hypertension, it had an inverse association with pre‐hypertension in our multivariable analysis. Additionally, education level had a positive association with hypertension. The higher prevalence and odds of hypertension among people with better socioeconomic conditions in developing countries primarily result from increased calorie consumption and sedentary lifestyles of these people compared with those with lower socio‐economic status.^40^ On the other hand, in developed countries, individuals with lower socio‐economic status have a greater risk of hypertension due to consumption of high‐calorie foods and having decreased awareness to control this condition.^30^, ^40^ We did not find any significant difference in prevalence or odds according to ecological zone or rural‐urban residence. However, the prevalence and odds of pre‐hypertension and hypertension were higher in specific regions, such as Provinces 4 and 5. This was also observed by another study that investigated risk factors for hypertension in Nepal.^41^ Regional variation has also been observed in studies from other countries, described primarily as a result of differences in socioeconomic conditions and dietary habits.^18^, ^42^, ^43^, ^44^ Similarly, substantial variations in health status have been observed in Nepal that result from ethnic variation, socioeconomic conditions, and dietary habits. Although the differences in prevalence or odds of hypertension were likely due to variations in dietary habits, ethnicity, and socioeconomic status in different regions that were beyond the scope of our analysis, another potential area for future investigation could be to determine the specific characteristics that may contribute to the higher prevalence and likelihood of high blood pressure levels found in Provinces 4 and 5.^45^, ^46^

Given the negative consequences of hypertension, our estimations suggest that hypertension could be a huge public health challenge for Nepal, specifically for some high‐risk groups such as older or obese people, similar to the situation in other countries.^12^, ^38^ The current health strategy has a specific target to reduce the prevalence of overall hypertension from 26% to 22% by 2020.^47^ Achieving this target could be too difficult for a country without a high awareness level. In Nepal, studies that investigated awareness of hypertension found a low level of awareness.^19^, ^41^ In addition, only a small proportion of people were taking anti‐hypertensive medications to control hypertension.^14^ These reduced awareness and treatment levels may put a large proportion of people at higher risks of cardiovascular diseases.

As stated previously, characteristics mainly associated with the development of pre‐hypertension and hypertension, such as lifestyle and dietary habits, were beyond the scope of this analysis. Nevertheless, the assessment of risk factor trends is imperative to understand varying prevalence and likelihoods among specific population groups. A recent study found that there is a trend towards sedentary lifestyle and the increased consumption of salty foods in a Nepalese community. These habits correlated with the prevalence of hypertension in that community.^16^ Future studies should investigate the overall trends of those characteristics and causal associations in this country.

The study has several notable strengths. First, we estimated the prevalence and determinants of pre‐hypertension and hypertension among a wide age range of people (≥15 years). This study is generalizable to the entire Nepalese population, as the survey covered both urban and rural areas in all seven provinces along with all three ecological zones. In addition, high response rate and a large sample size increased the reliability of the findings. Skilled survey staff used a standardized, validated method to measure the blood pressure of the participants.

Despite the above‐mentioned strengths, limitations of the present study also merit discussion. The 2016 NDHS dataset was cross‐sectional. Due to the uncertainty in temporality, associations observed in this study might not be causal. While the standard guidelines recommend longitudinal measurement of blood pressure with sphygmomanometers, the measurements were done with an automated device on a single day.^24^, ^26^ In addition, differences in the skill or efficacy level of survey staff may also have caused measurement errors.^18^ These variations could cause some non‐differential misclassification of disease. Due to limitations of the 2016 NDHS dataset, other factors such as ethnicity, physical activity level, concomitant diabetes, smoking, alcohol consumption, or dyslipidemia that also contribute to higher prevalence or likelihood of hypertension, were not investigated in our study; future studies should investigate association of these characteristics in the context of this country.

## 5 CONCLUSIONS

This study investigated the prevalence and determinants of pre‐hypertension and hypertension in Nepal. The combined prevalence of these two conditions indicates that nearly half of the people in this country have a higher risk of cardiovascular diseases due to elevated blood pressure. As a result, at‐risk individuals should adopt healthier dietary habits and more active lifestyles to prevent complications. People with certain background characteristics such as higher age, BMI, male sex, and residency in certain regions had increased prevalence and likelihood of pre‐hypertension and hypertension, and there is a need to prioritize these population groups in future hypertension control and prevention strategies.

## CONFLICT OF INTEREST

None declared.

## AUTHOR CONTRIBUTIONS

All authors have read and approved the final manuscript.

Conceptualization: Gulam Kibria

Formal analysis: Gulam Kibria, Muhammad Sakib

Investigation: Atia Sharmeen, Vanessa Burrowes, Krystal Swasey

Supervision: Vanessa Burrowes

Validation: Krystal Swasey, Muhammad Sakib

Visualization: Gulam Kibria

Writing—original draft preparation: Gulam Kibria, Atia Sharmeen

Writing—review and editing: Krystal Swasey, Muhammad Sakib, Vanessa Burrowes
